# Association Analysis of *GABRB3* Promoter Variants with Heroin Dependence

**DOI:** 10.1371/journal.pone.0102227

**Published:** 2014-07-15

**Authors:** Chia-Hsiang Chen, Chia-Chun Huang, Ding-Lieh Liao

**Affiliations:** 1 Department of Psychiatry, Chang Gung Memorial Hospital-Linkou, Taoyuan, Taiwan; 2 Department and Graduate Institute of Biomedical Sciences, Chang Gung University, Taoyuan, Taiwan; 3 Institute of Medical Sciences, Tzu-Chi University, Hualien, Taiwan; 4 Bali Psychiatric Center, Department of Health, Executive Yuan, Taipei, Taiwan; Yale University, United States of America

## Abstract

*GABRB3* encoding the β3 subunit of GABAA receptor has been implicated in multiple neuropsychiatric disorders, including substance abuse. Previous studies reported that SNPs at the 5′ regulatory region of *GABRB3* could regulate *GABRB3* gene expression and associated with childhood absence epilepsy (CAE). The study aimed to investigate whether SNPs at the 5′ regulatory region of *GABRB3* were associated with heroin dependence in our population. We first re-sequenced 1.5 kb of the 5′regulatory region of *GABRB3* gene to examine the SNP profile in the genomic DNA of 365 control subjects. Then, we conducted a case-control association analysis between 576 subjects with heroin dependence (549 males, 27 females) and 886 controls (472 males, 414 females) by genotyping the rs4906902 as a tag SNP. We also conducted a reporter gene assay to assess the promoter activity of two major haplotypes derived from SNPs at this region. We detected 3 common SNPs (rs4906902, rs8179184 and rs20317) at this region that had strong pair-wise linkage disequilibrium. The C allele of rs4906902 was found to be associated with increased risk of heroin dependence (odds ratio:1.27, p = 0.002). Two major haplotypes (C-A-G and T-G-C) derived from these 3 SNPs accounted for 99% of this sample, and reporter gene activity assay showed that haplotype C-A-G that contained the C allele of the tag SNP rs4906902 had higher activity than haplotype T-G-C. Our data suggest that *GABRB3* might be associated with heroin dependence, and increased expression of *GABRB3* might contribute to the pathogenesis of heroin dependence.

## Introduction

Heroin, a semi-synthetic form of morphine, has been considered to be one of the most addictive substances. Heroin dependence is a chronic, highly relapsed disease characterized by severe physical and psychological dependence. Genetic factor plays an important role in predisposing individuals to this disorder; the heritability of heroin dependence was estimated approximately 40–60% [1]. Heroin dependence is a complex disorder that brings a huge economic burden to the community [2]. Identification of susceptibility genes of heroin dependence would be helpful in understanding the neurobiology of heroin dependence and facilitate the treatment and the prevention of heroin dependence.

Several studies have provided evidence to indicate the involvement of gamma-aminobutyric acid (GABA) neurotransmission pathway in neurobiology of heroin dependence [3]. In an animal model of heroin dependence, relapse of heroin seeking was facilitated by extracellular matrix plasticity and GABAergic inhibition of prefrontal cortex pyramidal cells [4]. In addition, pharmacological studies showed that GABAB receptor agonists and its positive modulators were able to inhibit the reinforcing effects of drugs of abuse, such as cocaine, amphetamine, nicotine, ethanol, and opiates [5], [6]. In human subject study, a SNP rs211014 at *GABRG2* gene that encodes the γ2 subunit of GABAA receptor was reported to be associated with heroin dependence in a Chinese sample [7]. A recent study also reported that genetic polymorphisms of the glutamate decarboxylase 1 (GAD1) gene were associated with heroin dependence [8]. The GAD1 gene encodes the 67-kDa glutamic acid decarboxylase isoform (GAD67), and is the rate-limiting enzyme responsible for GABA biosynthesis from glutamic acid. Taken together, these findings support the involvement of GABAergic neurotransmission pathway in the pathogenesis of substance use disorders.

*GABRB3* gene encodes the β3 subunit of GABAA receptor that was mapped to chromosome 15q12 [9], a hot region of genomic rearrangements [10]. The region contains several paternal and maternal imprinted genes, and genomic rearrangements of this region are associated with neurodevelopmental disorders, such as Angelman syndrome, Prader-Willi syndrome [11], autism spectrum disorders [12] and schizophrenia [13]. The *GABRB3* gene has been considered a positional candidate gene of these neurodevelopmental disorders. Several previous studies reported that *GABRB3* was associated with alcoholism. Noble and colleagues studied the genetic association of dopamine D2 receptor gene (*DRD2*) and *GABRB3* with alcoholism, they found that variants of both *DRD2* and *GABRB3* contributed to the risk for alcoholism independently and when combined together, these two genes were robustly associated with alcoholism [14]. Song and colleagues reported that paternal transmission of *GABRB3* was associated with alcoholism [15]. Young and colleagues reported that *DRD2* and *GABRB3* were associated with alcohol-related expectancies [16]. The association between *GABRB3* and heroin dependence has not yet been well addressed in the literature. A recent genetic study reported that a SNP (rs7165224) located at proximity of the *GABRB3* gene was nominally associated with heroin addiction in a sample of African Americans [17], suggesting *GABRB3* gene might be also associated with heroin dependence.

Rare genetic missense mutations of *GABRB3* have been found to segregate with childhood absence epilepsy (CAE) in some families [18]–[22]; hence, *GABRB3* was considered a Mendelian gene for CAE. Further, studies showed that several SNPs were present at the 5′ regulatory region of *GABRB3*, and some of them could influence the expression of *GABRB3* [20], [22]. Urak and colleagues reported that a haplotype 2 promoter of *GABRB3* that caused reduced reporter gene activity was associated with CAE. Thus, they suggested that reduced *GABRB3* expression might be involved in the pathogenesis of CAE [22]. Prompted by these findings, we were interested to know whether functional variants at the 5′ regulatory region of *GABRB3* were associated with heroin dependence in our population. To address this issue, we first re-sequenced 1.5 kb of the 5′ region of *GABRB3* in a sample of control subjects to examine the SNP profile at this region, as different populations may have different SNP profiles. Then we conducted a case-control association analysis of heroin dependence by genotyping a tag SNP at this region. Finally, we conducted a reporter gene activity assay to characterize the functional impact of two haplotypes derived from SNPs at this region.

## Materials and Methods

### Subjects

All the subjects in this study were Han Chinese from Taiwan. A total of 576 patients (549 males, 27 females, mean age ± standard deviation  = 37.4±9.6 years) meeting the diagnostic criteria of heroin dependence as defined by DSM-IV were recruited from Bali Psychiatric Center, Taiwan. The diagnosis of heroin dependence was made based on medical records and interviews by a senior psychiatrist. Subjects with co-morbidity of other DSM-IV axis I psychiatric diagnoses were excluded from this sample. A total of 886 non-psychiatric control subjects (472 males, 414 females, mean age ± standard deviation = 43.5±15.8 years) were recruited from the Department of Family Medicine of Buddhist Tzu Chi General Hospital, Hualien, Taiwan. The mental status and history of mental illness of the control subjects were evaluated by a senior psychiatrist using the Mini-International Neuropsychiatric Interview (MINI) [23]. Subjects diagnosed with a DSM-IV axis I disorders including substance use disorders such as alcohol, nicotine, and illicit drugs were excluded. The study protocol was approved by the Ethics Committee of Bali Psychiatric Center, Taiwan and Buddhist Tzu Chi General Hospital, Hualien, Taiwan. Written informed consent was obtained after full explanation of the protocol. For heroin dependent individuals, written informed consent was obtained from both participants and their parents (or legal guardians) after full explanation of the protocol and reassurance of confidentiality and voluntary participation.

### Direct sequencing of the 5′ regulatory region

Genomic DNA was extracted from peripheral blood using the Gentra Puregene Blood kit according to the manufacturer's instructions (Qiagen, Hilden, Germany). We designed 3 PCR amplicons to cover 1.5 kb of the 5' region of GABRB3 (NM_021912.4). The primer sequences, optimal PCR conditions, and the size of amplicons are listed in **Table S1**. In a standard PCR, genomic DNA (100 ng) was amplified in a reaction volume of 15 µl containing 1 µM each of sense and antisense primer, 0.2 mM of dNTP, 50 mM of KCl, 1.5 mM of MgCl_2_, 0.1% vol/vol of Triton X-100, 10 mM of Tris-HCl (pH 9.0), and 2.5 U Taq polymerase. PCR cycling conditions consisted of an initial denaturation at 95°C for 10 min, followed by 30 cycles of 95°C for 1 min, an annealing temperature 60°C for each amplicon for 1 min, and 72°C for 1 min. After PCR amplification, aliquots of PCR products were processed using a PCR Pre-Sequencing Kit (USB Corp., Cleveland, OH) to remove residual primers and dNTPs. These purified PCR products were then subjected to direct sequencing using an ABI PRISM BigDye Terminator Cycle Sequencing Ready Reaction Kit Version 3.1, and an ABI autosequencer 3730 (Perkin Elmer Applied Biosystems, Foster City, CA), according to the manufacturer's protocol.

### Restriction fragment length polymorphism (RFLP) analysis

For genotyping of the tag SNP rs4906902, aliquots of PCR product were digested with 1 unit of AseI overnight at 37°C incubation, then subjected to 2% agarose gel electrophoresis. The T allele yielded 262 and 427 bp PCR fragments, while the C allele remained uncut (689 bp). The reader of RFLP results was blind to the diagnosis. For quality assurance, 10% of the subjects were randomly selected for Sanger sequencing. The concordance rate between RFLP and PCR-based sequencing was 100%.

### Reporter gene assay

Genomic DNAs were used to construct the inserts for the reporter gene assay. A sense primer containing the KpnI recognition site linker (5′- gaatctttcaggtactgcggtca-3′) and an antisense primer containing the XhoI recognition site linker (5′-ctgttcctccggcctaacct-3′) were used to PCR amplify the fragment from nucleotide positions -4 bp to -979 bp upstream to the ATG starting nucleotide of exon 1a of the GABRB3 gene. The PCR fragments were first cloned into a pCR-Blunt II-TOP vector (Invitrogen, CA, USA), then subcloned into the pGL3-enhancer vector (Promega, Madison, WI, USA); the authenticity of these clones was verified by sequencing. Plasmids were transfected into a SK-N-SH (ATCC HTB-11) neuroblastoma cell line (American Type Culture Collection, Manassas, VA USA) cultured in Minimum Essential Medium (MEM) containing 10% fetal bovine serum in 24-well plates using LipofectamineTM2000 (Invitrogen, California, USA) according to the manufacturer's protocol. Each well contained 10^5^ cells, 800 ng of reporter plasmid, 200 ng of pRL-TK (Promega, Madison, WI, USA) as an internal control, and 2 µl of LipofectamineTM2000. At 30 hours after transfection, cells were lysed and the luciferase activities were measured using the Dual-Luciferase Reporter Assay System according to the manufacturer's instructions (Promega, Madison, WI, USA). The firefly luciferase activity was normalized against the Renilla luciferase activity in each transfection. The promoter activity of each plasmid was assayed in triplicates, and the promoter driving activity of haplotypes C-A-G and T-G-C relative to that of pGL3-enhancer vector was calculated as relative fold change.

### In silico analysis

An online software WWW Signal Scan (http://www-bimas.cit.nih.gov/molbio/signal/) was used to predict the alteration of putative transcription factor binding sites influenced by genetic variants.

### Statistical analysis

Deviation from the Hardy-Weinberg equilibrium of the genotype distribution in both the patient and control groups was determined by Chi-square test. Genetic Power Calculator (http://pngu.mgh.harvard.edu/~purcell/gpc/) was used to perform a post-hoc power analysis. Linkage disequilibrium (LD) analysis was performed using Haploview version 4.2 [24]. Differences in allele, genotype, and estimated haplotype frequencies between patients and controls were evaluated using SHEsis [25]. Reporter gene activity between 2 constructs was compared using *t*-test. A p-value of less than 0.05 was considered significant.

## Results

### Detection of common variants and LD analysis

**Figure 1:**
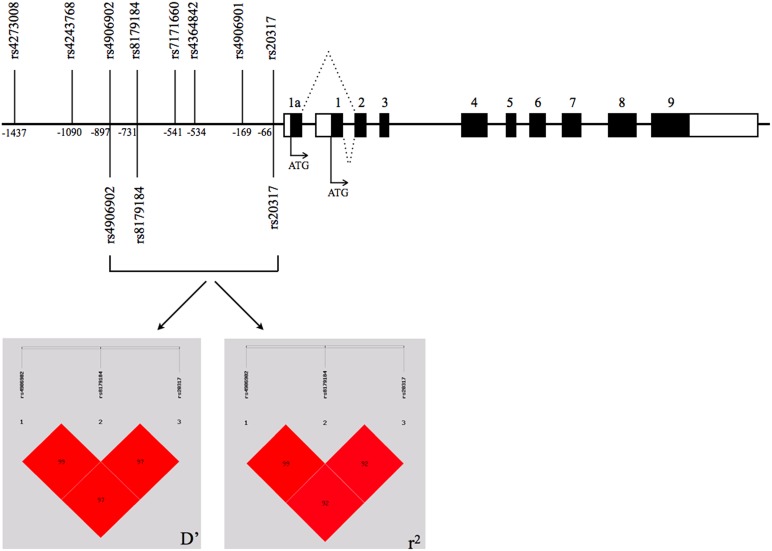
Schematic genomic structure of the 5′ region of GABRB3 gene and LD plot of 3 SNPs identified in this study. The shaded boxes represent the protein-coding regions, while the open boxes represent the untranslated regions. SNPs reported in the paper published by Urak and colleagues [22] were displayed at the upper panel of the schematic *GABRB3* genomic structure for comparison with our findings that were listed at the low panel of the schematic *GABRB3* genomic structure. The positions of the SNPs are numbered from the ATG start site of exon 1a. Dotted lines indicate alternative splicing of the first exons. The numbers in LD blocks are D′ and r^2^ values.

After re-sequencing 1.5 kb of the genomic DNA upstream of exon 1a in 386 normal subjects, we detected three SNPs (rs4906902, rs8179184, and rs20317) that have been reported in the National Center for Biotechnology Information (NCBI) SNP database (http://www.ncbi.nlm.nih.gov/snp/). These 3 SNPs had strong pair-wise linkage disequilibrium to each other. The genotype and allele frequency of these 3 SNPs are listed in **Table 1**. Positions of these 3 SNPs and their derived haplotype block are shown in **Figure 1**. The D' values between rs4906902 and rs8179184, between rs8179184 and rs20317, and between rs4906902 and rs20317, were 1.00, 0.98, and 098, respectively, while the r^2^ values were 0.98, 0.96, and 0.96, respectively. Four estimated haplotypes derived from these 3 SNPs, including C-A-C, C-A-G, T-G-C, and T-G-G. Haplotypes T-G-C (63.6%) and C-A-G (35.4%) were two major haplotypes that accounted for 99% of the sample, while C-A-C (0.5%) and T-G-G (0.3%) were minor haplotypes that counted for less than 1% of the sample.

**Table 1:** The genotype and allele frequency of three SNPs identified in 386 control subjects in his study.

SNP	Allele (1/2)	Genotype counts (frequency)			Allele counts (frequency)	
		1/1	1/2	2/2	1	2
rs4906902	T/C	161 (0.42)	171 (0.44)	54 (0.14)	493 (0.64)	279 (0.36)
rs8179184	G/A	162 (0.42)	171 (0.44)	53 (0.14)	495 (0.64)	277 (0.36)
rs20317	C/G	162 (0.42)	172 (0.45)	52 (0.14)	496 (0.64)	276 (0.36)

### Genetic association study

**Figure 2:**
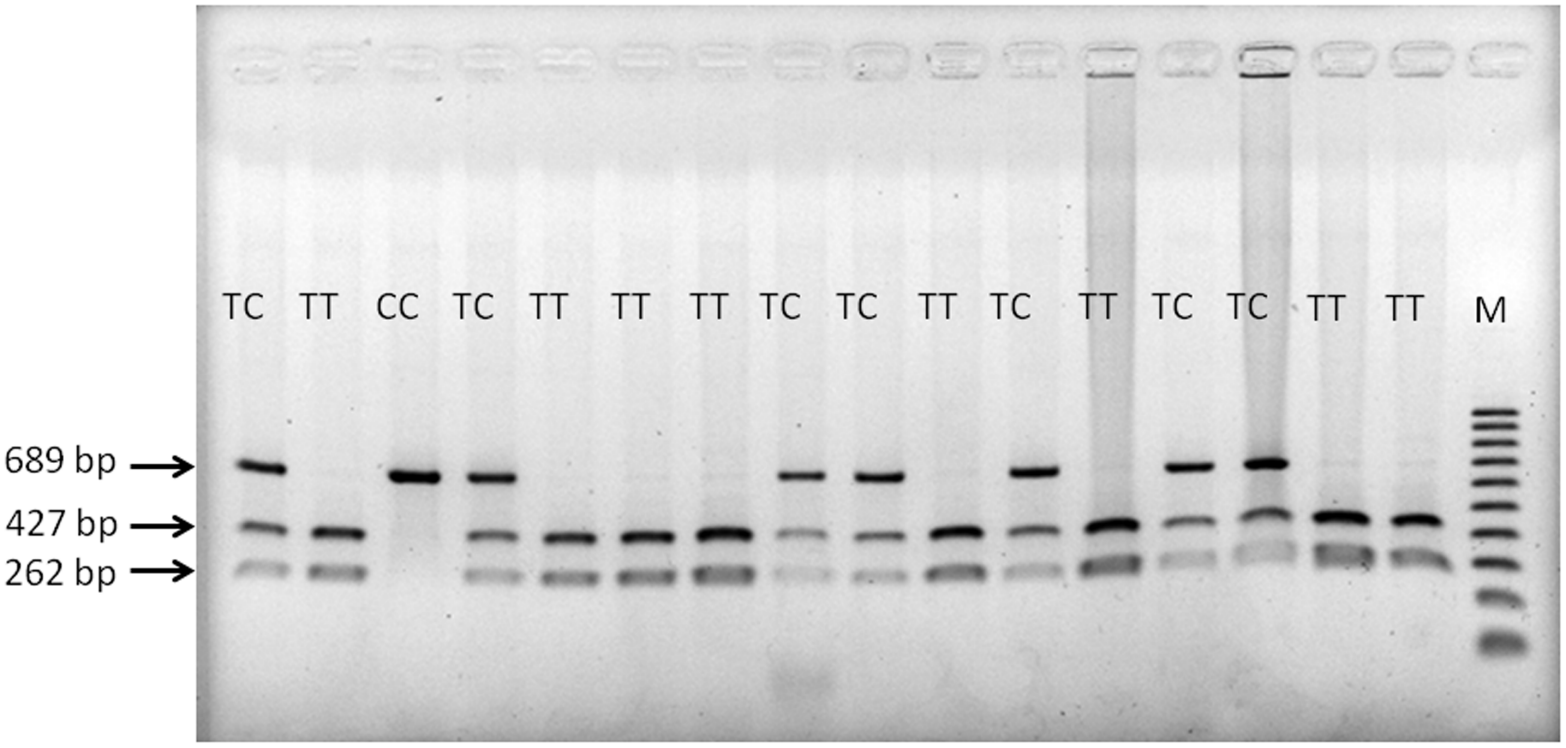
A representative genotyping results of rs4906902 using RFLP. After digestion with AseI, the T allele yielded 262 and 427(689 bp); M indicates DNA marker 100 bp ladder.

Since these 3 SNPs were in strong LD, we selected rs4906902 as the tag SNP and genotyped this SNP in 576 patients with heroin dependence and additional 500 control subjects using RFLP. The representative RFLP results of rs4906902 are illustrated in **Figure 2**. The allelic and genotypic frequencies distributions of rs4906902 are listed in **Table 2**. The distributions of genotypic frequencies did not deviate from Hardy-Weinberg equilibrium in both case and control groups. We detected significant differences in the genotypic (p = 0.008) and allelic (p = 0.003) frequencies between the patient and the control groups. The C allele of rs4906902 was significantly over-represented in the patient group (odds ratio of 1.27, 95% confidence interval: 1.09–1.49, p = 0.002). As most of our patients were males (95%), we further analyzed the association based on gender. The association of the C allele of rs4906902 with heroin dependence in males became marginal (p = 0.07), and was not significant in female subjects (**Table 2**).

**Table 2:** Association analysis of rs4906902 with heroin dependence.

Variant	Group	n	Genotype counts (frequency)			p	Allele counts (frequency)		p
rs4906902			C/C	C/T	T/T	0.008	C	T	0.003
Total	Drug	576	98 (0.17)	256 (0.45)	222 (0.39)		452 (0.39)	700 (0.61)	
	Control	886	101 (0.11)	394 (0.45)	391 (0.44)		596 (0.34)	1176 (0.66)	
rs4906902			C/C	C/T	T/T	0.135	C	T	0.067
Male	Drug	549	95 (0.17)	244 (0.44)	210 (0.38)		434 (0.40)	664 (0.60)	
	Control	472	61 (0.13)	214 (0.45)	197 (0.42)		336 (0.36)	608 (0.64)	
rs4906902			C/C	C/T	T/T	0.955	C	T	0.767
Female	Drug	27	3 (0.11)	12 (0.44)	12 (0.44)		18 (0.33)	36 (0.67)	
	Control	414	40 (0.10)	180 (0.43)	194 (0.47)		260 (0.31)	568 (0.69)	

### In silico analysis and reporter gene activity assay

**Figure 3:**
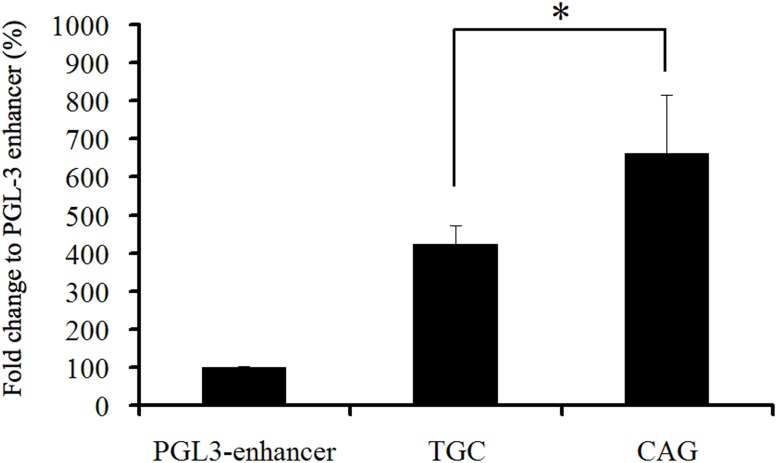
Reporter gene activity assay of two haplotypes of GABRB3 in this study. The haplotype C-A-G had a significantly higher promoter activity than haplotype T-G-C. The pGL-3 enhancer was an empty vector that did not contain the insert of interest, whose activity was used as background activity in this study. (* indicates p<0.01).

In silico analysis predicted that these 3 SNPs changed the transcription factor binding sites (**Table 3**). As haplotypes C-A-G and T-G-C accounted for the majority (99%) of the sample, we conducted a reporter gene activity assay of these two haplotypes. As shown in **Figure 3**, the haplotype C-A-G that contained the C allele of the tag SNP rs4906902 had significantly higher reporter gene activity than the haplotype T-G-C (∼1.5 fold, p<0.05).

**Table 3:** Predicted changes of transcription factor binding sites of 3 SNPs identified in this study.

dbSNP ID	Allele location	Putative regulatory element binding
rs4906902	c.-897T>C	T allele: GMCSF CS; C allele: none
rs8179184	c.-731G>A	G allele: none; A allele: gamma-IRE CS
rs20317	c.-66C>G	C allele: myosin-specific factor; G allele: none

## Discussion

In this study, after re-sequencing 1.5 kb of the 5′ regulatory region of *GABRB3* gene in a sample of control subjects, we detected only three common SNPs (rs4906902, rs8179184, and rs20317) in our sample. We did not detect the other SNPs that were reported in the SNP database or in the literature, such as rs4273008, rs4243768, rs9672674, rs7171660, rs4363842, rs112488959, and rs4906901 [20], [22]. We also did not detect the presence of rs7165224 in our sample that had a minor allele frequency (MAF) of 0.254 in African Americans [17]. Furthermore, we found two SNPs (rs4906902 and rs8179184) that were common in our sample were extremely rare in Africans according to the NCBI SNP database. These data suggest that there might have different SNP profiles at the 5′ regulatory region of the *GABRB3* gene across different populations. Due to the population stratification of SNPs at the 5′ regulatory region of *GABRB3*, the haplotypes found in our sample were also different from those reported in the literature. Of note, it is likely that there might be variants located at the region beyond 1.5 kb of the 5′ regulatory region of GABRB3, which will be missed by this study as we only sequenced 1.5 kb of the 5′ regulatory region of *GABRB3*.

In this study, we found the tag SNP rs4906902 was associated with heroin dependence. However, due to the small effect size and sample size, post-hoc genetic power analysis revealed that this study had only 57% power to detect the association under the assumptions of multiplicative inheritance mode, genotype relative risk = 1.2, prevalence of disease = 0.01 and alpha level = 0.05. This is a major limitation of this study. In addition, when the subjects were stratified based on gender, the association of the tag SNP rs4906902 with heroin dependence in males became marginal, and not significant in females, which might be due to the small sample size of the present study. Hence, our data can only be considered as preliminary; further replications studies with larger sample are needed to verify our findings in this study.

Previous studies had reported that *GABRB3* was associated with several neuropsychiatric disorders, such as such as Angelman syndrome, Prader-Willi syndrome, autism spectrum disorders, schizophrenia, and alcoholism. To avoid these confounders, we have excluded those subjects with co-morbidity of other axis I psychiatric diagnoses in this study as much as we can. Hence, if the association between *GABRB3* gene and heroin dependence was further supported by other research groups, the *GABRB3* gene could be considered as a shared susceptible gene among these neuropsychiatric disorders, not limited to heroin dependence.

Previous studies reported that SNPs at the 5′ regulatory region of the *GABRB3* gene and their derived haplotypes had different promoter activities [20], [22]. Urak and colleagues reported that a *GABRB3* promoter haplotype with reduced transcription activity was associated with childhood absence epilepsy [22]. In their study, they conducted reporter gene activity assays using NT2 cells, a human neuroblastoma cell line. In this study, we conducted reporter gene assays in another human neuroblastoma cell line, SK-N-SH (ATCC HTB-11) purchased from American Type Culture Collection (Manassas, VA USA). We demonstrated that the haplotype C-A-G that contained the C allele of rs4906902 had significantly higher reporter gene activity than the haplotype T-G-C. As the C allele of haplotype C-A-G was the risk allele associated with heroin dependence in this study, our data suggested that increased *GABRB3* gene expression might contribute to the pathogenesis of heroin dependence.

Although the real mechanism underlying the association remains to be investigated, we infer that mechanistically increased *GABRB3* gene expression may lead to increased GABAA receptor numbers in the synaptic surface of the dopaminergic neuron and which results in enhanced synaptic inhibition [26]–[28]. Enhanced GABAergic inhibition may in turn lead to attenuation of dopaminergic neuron activity, which has been reported to be associated with increasing susceptibility to substance abuse [29], [30]. Thus, we speculate that the C allele that associated with heroin addiction may lead to hypodopaminergic activity in the rewarding circuit and increase the propensity to substance abuse, such as heroin in this study.

In conclusion, our data suggest that *GABRB3* gene might be associated with heroin dependence, and increased *GABRB3* gene expression might contribute to the pathogenesis of heroin dependence. However, our study is limited by the small sample size; hence, replication studies with a larger sample size are needed to verify our findings. As functional GABAA receptor is protein complex that consists of multiple subunits, it is worthwhile to examine the association of the other subunit genes of GABAA receptor with heroin dependence in the future.

## Supporting Information

Table S1 **Primer sequences, optimal annealing temperature (Ta), and size of PCR products for PCR amplification of the 5′ regulatory region of the GABRB3 gene.**
(DOCX)
