# Supplementary material for: Association Analysis of GABRB3 Promoter Variants with Heroin Dependence
Source: PLoS One. 2014 Jul 15;9(7):e102227. doi: 10.1371/journal.pone.0102227 (PMC4098998; doi:10.1371/journal.pone.0102227)
Supplement: Table S1 — Primer sequences, optimal annealing temperature (Ta), and size of PCR products for PCR amplification of the 5′ regulatory region of the GABRB3 gene. (DOCX) [file pone.0102227.s001.docx]

**Table S1.** Primer sequences, optimal annealing temperature (Ta), and size of PCR products for PCR amplification of the 5’ regulatory region of the GABRB3 gene

| Amplicon | Forward (5’-3’) | Reverse (5’-3’) | Ta (^○^C) | Size (bp) |
| --- | --- | --- | --- | --- |
| Exon 1a promoter1 | TCACAGGATCATCTTTGAGAGG | TGTGACCGCAGTACCTGAAA | 60 | 693 |
| Exon 1a promoter2 | CAGGGCATTTCTCCAAAAGA | TAGAAGGCTACTGGCGCACT | 60 | 689 |
| Exon 1a promoter3 | GAACACAAAAACGAGCTTGATG | GGTCCAGGAGAGCCAGATG | 60 | 769 |
